# Dietary intakes of trans fatty acids before the prohibition of partially hydrogenated oils in Canada

**DOI:** 10.1007/s00394-024-03569-7

**Published:** 2024-12-30

**Authors:** Isabelle Demonty, Kuan Chiao Wang, Isabelle Rondeau, Chantal Martineau, Lindsay Manley, Janice Daoust, Kathryn Hopperton

**Affiliations:** 1 Nutrition Research Division, Bureau of Nutritional Sciences, Health Products and Food Branch, Health Canada, 251 Sir Frederick Banting Driveway, Ottawa, ON K1A 0K9 Canada; 2 Bureau of Data, Science and Knowledge Integration, Health Products and Food Branch, Health Canada, Ottawa, ON Canada; 3 Nutrition Regulations and Standards Division, Bureau of Nutritional Sciences, Health Products and Food Branch, Health Canada, Ottawa, ON Canada

**Keywords:** *Trans* fatty acids, Dietary intakes, Industrially produced *trans* fatty acids, Partially hydrogenated oils, Naturally occurring *trans* fatty acids, Canadian Community Health Survey

## Abstract

**Purpose:**

Canada’s public health objective is that ≥ 90% of the population consume <1% of total energy (< 1%En) as *trans* fatty acids (TFA), in line with World Health Organization recommendations. Our study aimed to estimate usual intakes of total TFA, industrially-produced TFA (i-TFA), and naturally occurring TFA (n-TFA) overall and in subgroups of the population before Canada’s 2018 prohibition on the use of partially hydrogenated oils (PHO) in foods.

**Methods:**

Data from 1–2 24-h recalls was available for 19,670 participants in the cross-sectional Canadian Community Health Survey (CCHS)—Nutrition 2015. Usual intakes of total TFA, i-TFA, n-TFA, and mixed TFA (TFA from foods containing both i-TFA and n-TFA) from all foods and beverages were generated according to the National Cancer Institute method, and weighted to represent the population of Canada aged ≥ 1 and within age, sex, income, and self-reported racial groups.

**Results:**

For the overall population, the mean usual intake of total TFA was 1.2 g/day (SE:0.02) and represented 0.57%En (SE:0.001). All age-sex groups had mean total TFA intakes <1%En, ranging from 0.52 to 0.71%En. On average, foods containing only n-TFA provided >1/2 of total TFA intake (0.32%En, SE:0.01). The target of ≥ 90% of the population consuming <1%En as TFA had already been achieved before the PHO prohibition in all income, racial, and age-sex groups, except children 1–3 years old, with 86% within target. In that group, foods containing only n-TFA provided >2/3 of total TFA intake (0.48%En, SE:0.02).

**Conclusion:**

Total TFA intakes in Canada before the PHO prohibition were relatively low, likely due to previous initiatives to reduce i-TFA in foods.

**Supplementary Information:**

The online version contains supplementary material available at 10.1007/s00394-024-03569-7.

## Introduction

Trans-fatty acids (TFAs) represent a category of unsaturated fatty acids characterized by the presence of double bond(s) in the *trans* configuration. They occur naturally, in foods such as meat and dairy from ruminant animals, and are also produced through heating or industrial processing, such as in the production of partially hydrogenated oils (PHO), which may be added to foods as a low-cost alternative to other fat sources, or to extend shelf life [1]. Both naturally-occurring (n-TFA) and industrially-produced (i-TFA) sources of TFA consist of the same molecules in different amounts and proportions, with PHO containing up to 60% of total fat as TFA, compared to 0.5–8% of total fat in foods from ruminant animals [1, 2]. World Health Organisation (WHO) guidelines, updated in 2023, recommend limiting intakes of total TFA, including both industrial and ruminant sources, to 1% of total daily energy (%En) on the basis of moderate evidence for lower LDL cholesterol, and risks of cardiovascular disease (CVD), coronary heart disease (CHD) and all cause mortality [3]. There is also a second conditional recommendation to reduce TFA intakes below 1%En based on low certainty of evidence. In support of earlier versions of these recommendations, the WHO released the REPLACE action package in 2018, which aimed to help countries reduce intakes of TFA through the elimination of i-TFA from the food supply by 2023 [4].

In the early 2000s, Canada initiated strategies to lower intakes of i-TFA. These included mandatory declaration of *trans* fat in the Nutrition Facts table on food labels, the establishment of a *Trans* Fat Task Force, voluntary targets for TFA content in foods (≤2% of fat for oils and soft spreadable margarines; and <5% of fat for other foods), and monitoring and open reporting on industry’s progress toward meeting the voluntary targets for a 2-year period from 2007 to 2009 [5, 6]. The combined impact of these strategies led to product reformulations and significant reductions in the TFA content of many foods, with 97% of foods meeting the targets by 2010–11 [6, 7]. However, some food categories continued to have large proportions of foods exceeding targets, and the Canadian government’s risk assessment of exposure to TFA concluded that certain population subgroups continued to be at risk of high TFA intakes, including children and adolescents, low-income subgroups, and people living in remote areas, such as Inuit populations in Nunavik [5, 6].

As part of its Healthy Eating Strategy and to align with WHO recommendations, Canada introduced a prohibition on the use of PHO in foods in September 2018, with a 2-year phase-in period such that as of September 2020, no foods sold in Canada are permitted to contain PHO [8]. To measure the performance of this initiative, along with other components of its Healthy Eating Strategy, Health Canada set a target to have ≥ 90% of the population with total TFA intakes below 1%En by the end of 2023 [9].

The most recent assessment of TFA intakes of people in Canada used 2004 dietary intake data and measurements of TFA food content collected from 2004 to 2008. Results showed that people consumed on average 1.4%En (3.4 g/day) as TFA, thus exceeding the WHO recommendation [5]. However, the data used on dietary intakes and TFA food content predated the establishment of some of the Canadian *trans* fat reduction initiatives of the early 2000s, and did not account for potential changes in consumer behavior with improved awareness of the presence and health effects of TFA and for the full extent of product reformulation by industry in response to government policies. A new, more recent assessment of baseline TFA intakes of people in Canada is needed to evaluate the impact of the 2018 PHO prohibition [5]. The aim of our study was to determine intakes of total TFA, n-TFA and i-TFA using recent food composition data along with data from the Canadian Community Health Survey (CCHS) Nutrition 2015 and to identify food categories that contribute most to total and i-TFA intakes.

## Subjects and methods

### The Canadian Community Health Survey-Nutrition

The 2015 CCHS Nutrition is a nationally representative, cross-sectional health survey in Canada conducted from January to December 2015, which provides detailed information at the national and provincial levels on food consumption, including estimated distribution of usual dietary intake in terms of nutrients from food [10]. Participants included those aged ≥ 1 year living in private dwellings in the ten Canadian provinces, excluding those living in the Territories, full-time members of the Canadian Forces and individuals living on reserves and other Aboriginal settlements, in some remote areas, or in institutions. Respondents were interviewed using a computer assisted in-person interview. In most cases, primary interviews were conducted in person, and were completed in participants’ homes. The interview included a 24-h diet recall as well as collecting information that would support interpretation of the data from the 24-h recall, such as anthropometric measurements and socio-economic and demographic characteristics. The 24-h recall asked participants to recall all foods and beverages consumed the preceding day, including packaged and unpackaged foods and dishes consumed in or outside of the home. During the first interview, a random subset of individuals was invited to take part in a second interview approximately 3 to 10 days after the first interview, on a different day of the week. This second interview was conducted by telephone and included only a 24-h recall and questions about nutrient supplements. Food recalls were conducted according to an adapted Automated Multiple-Pass Method as previously described [10, 11]. For children aged 1 to 5 years, the parent or guardian was asked to provide the child's information, and for respondents aged 6 to 11 years, the child was asked to provide their information with the help of a parent or guardian. In total, 20,487 individuals took part in the first 24-h dietary recall, and 7,623 in the second 24-h recall (see flow diagram Supplemental Fig. 1). The response rate was 61.6% for the first 24-h recall and 68.6% for the second [11, 12]. Data from participants who did not consent to share data with partners (n = 814) was unavailable for this analysis, leaving a total sample size of 19,673 [13]. Participation in the survey was voluntary, and data were collected in accordance with the Statistics Act of Canada. No separate ethical approval was required for the present analysis.

### Categorization of population subgroups

Twelve age-sex groups were defined to align with those used in the Dietary Reference Intakes (DRIs): 1 to 3 years (sexes combined), 4 to 8 years (sexes combined), 9 to 13 years (males and females), 14 to 18 years (males and females), 19 to 50 years (males and females), 51 to 70 years (males and females), and ≥ 71 years (males and females) [14]. Data were also analyzed by race and income to identify sub-populations who may be at disproportionate risk of high TFA intake. Self-reported race was categorized according to the Canadian Institute of Health Information Standards for race-based data reporting and grouped as Black, East Asian (Chinese, Korean, Japanese), Indigenous (First Nations, Inuk/Inuit, Métis), Latin American, Middle Eastern (Arab, West Asian), South Asian (e.g. East Indian, Pakistani, Sri Lankan), Southeast Asian (e.g. Filipino, Vietnamese, Cambodian, Laotian, Thai), White, and Other (other not included in previous categories, multiple racial identities, did not know, and preferred not to answer) [15]. Total household income was categorized in six groups of relatively similar size (number of participants): less than $30,000, $30,000 to $49,999, $50,000 to $69,999, $70,000 to $99,999, $100,000 to $149,999, and $150,000 or more.

### TFA composition and categorization of foods

A nutrient database was developed for the 2015 CCHS Nutrition using three data sources: the 2015 Canadian Nutrient File (CNF, a food composition database that includes analytical data on nutrients in > 5000 foods available in Canada), a Canadian recipe file based on the U.S Department of Agriculture Food and Nutrient Database for Dietary Studies, and survey foods (foods not present in the other data sources) as described [16]. While the majority (> 70%) of foods consumed in the 2015 CCHS nutrition had data available from these sources, additional data was obtained from food composition label data collected by Nielsen in 2016 to account for product reformulations in recent years [17]. The remaining foods used from the CNF and other sources were either natural sources of TFA that were considered unlikely to have substantially changed since their measurement (e.g. dairy products, meats), or foods that provided no or negligible amounts of TFA. Label data were used for the following pre-packaged food categories, which were identified as main contributors to i-TFA intakes in a preliminary assessment using CCHS-Nutrition 2004 data: bakery products (21 sub-categories); breakfast cereals (3 subcategories); snacks (6 sub-categories); fats, oils & dressings (6 sub-categories); mixed dishes (12 sub-categories); nut butters (1 sub-category); sauces, dips, gravies and condiments (7 sub-categories); soups (3 sub-categories); confectionary (3 sub-categories); coffee whiteners (1 sub-category). For each food sub-category, the sales weighted average of TFA content was determined. These values were then applied to the applicable foods and recipes in the 2015 CCHS Nutrition [11]. Overall, < 2% of foods reported to be consumed in the 2015 CCHS Nutrition were missing data on TFA content.

Health Canada had previously matched pre-packaged food composition label data to the foods and beverages from the 2015 CCHS Nutrition for its sodium reduction programs [11, 18]. This pre-packaged food composition label data was used as a starting point to reflect main contributions to Canadian TFA intakes (based on data from the CCHS Nutrition 2004), by grouping foods with similar TFA levels based on the distribution of TFA content per 100 g. Additional Nielsen data was purchased for the following categories of pre-packaged foods that were identified as main contributors to TFA intakes, but were not included in the label data purchased for the sodium reduction program: chocolate, baking chips, baking chocolate solid, coffee whiteners, ice cream cones, lard & shortening. This resulted in 48 subgroups within sixteen main categories: (i) Cookies, baked desserts, pastries & confectionery, (ii) Snack foods, (iii) Breads, wraps & rolls, (iv) Milk & yoghurt, (v) Butter, (vi) Cheese, (vii) Ice cream and frozen yoghurt, (viii) Cream, (ix) Beef, (x) Sausage, (xi) Other meats, (xii) Mixed dishes, (xiii) Fats, oils and dressings, (xiv) Sauces, dips, gravies, and condiments, (xv) Cereals and ready-to-eat breakfast, and (xvi) Other foods and beverages (See Supplemental Table 1 for list of all subgroups).

Supplemental Table 1 shows the categories and sub-groups used. TFA-containing foods were further categorized as i-TFA (containing only industrial sources of TFA), n-TFA (containing only naturally occurring sources of TFA) or mixed (containing both sources).

### Determination of usual intakes and statistical analysis

All analyses were conducted in SAS version 9.3 or the SAS-callable SUDAAN version 11.0.1, and applied survey sampling weights to account for the complex sampling design and unequal probability of selection so that results are generalizable to the population in Canada. Five hundred bootstrap weights provided with the survey were used to calculate robust standard errors and coefficients of variation.

Usual (i.e., habitual or typical) intakes of total TFA, i-TFA, n-TFA and mixed TFA (TFA from foods containing both i-TFA and n-TFA) were calculated for the overall population and for the different population subgroups described above according to the National Cancer Institute (NCI) method [11, 19–21]. Total TFA used a 1-part NCI model, as almost all participants had TFA intakes, while i-TFA, n-TFA and mixed TFA were estimated using 2-part models as they were more episodically consumed. Intakes were expressed in g TFA/day and in percentage of total daily energy intake (%En).

## Results

### Usual intakes of total TFA, TFA from foods containing i-TFA only, TFA from foods containing n-TFA only, and TFA from foods containing mixed TFA

For the overall population, the mean usual intake of total TFA was 1.19 (SE 0.015) g/day and represented 0.57 (SE 0.001) %En. (Table 1). When expressed in %En, total TFA intakes appeared to be higher in children aged up to 13 years (0.64 [SE 0.031] to 0.71 [0.022] %En) than in the other age-sex groups, in which total TFA intakes were at or below 0.61% (Table 1). The 90th percentiles of total TFA intake were below the WHO threshold in the total population and in all age-sex groups except for children aged 1–3 years (90th percentile 1.07 [SE 0.061] %En) (Table 2). Among children aged 1–3 years, 85.9% were below the threshold. The 95th percentile of total TFA intakes was also below the WHO threshold in the total population and all groups except children aged 1–3 years (1.2 [SE 0.078] %En), males aged 31–50 (1.01 [SE 0.063] %En), and males aged ≥ 71 years (1.01 [SE 0.062] %En) (Table 2).

**Table 1:** Mean usual intakes of total TFA, TFA from foods containing only industrially produced TFA (i-TFA), TFA from foods containing only naturally occurring TFA (n-TFA), and TFA from foods containing a mix of i-TFA and n-TFA by people in Canada aged 1 year and older before the prohibition of partially hydrogenated oils

Study population											
		n	%	Total TFA		i-TFA		n-TFA		Mixed TFA^b^	
				g/day	% Energy	g/day	% Energy	g/day	% Energy	g/day	% Energy
				Mean (SE)	Mean (SE)	Mean (SE)	Mean (SE)	Mean (SE)	Mean (SE)	Mean (SE)	Mean (SE)
Total		19,670	100	1.19 (0.02)	0.57 (0.001)	0.44 (0.01)	0.21 (0.01)	0.66 (0.01)	0.32 (0.01)	0.18 (0.004)	0.08 (0.002)
*Age and sex groups*											
1–3 y		1288	3.3	1.06 (0.05)	0.71 (0.02)	0.3 (0.03)	0.20 (0.02)	0.71 (0.04)	0.48 (0.02)	0.02E (0.01)	0.01E (0.03)
4–8 y		1199	5.5	1.23 (0.05)	0.66 (0.01)	0.53 (0.03)	0.28 (0.02)	0.61 (0.02)	0.33 (0.01)	0.17 (0.01)	0.09 (0.004)
9–13 y	Males	1023	2.7	1.57 (0.08)	0.68 (0.04)	0.69 (0.07)	0.31 (0.03)	0.74 (0.03)	0.33 (0.01)	0.18 (0.02)	0.08 (0.03)
	Females	939	2.6	1.34 (0.06)	0.64 (0.03)	0.55 (0.05)	0.27 (0.02)	0.68 (0.03)	0.33 (0.01)	0.17 (0.01)	0.08 (0.05)
14–18 y	Males	900	2.9	1.64 (0.08)	0.59 (0.02)	0.61 (0.06)	0.22 (0.02)	0.84 (0.05)	0.31 (0.02)	0.28 (0.02)	0.12 (0.05)
	Females	990	2.7	1.20 (0.06)	0.58 (0.03)	0.45 (0.04)	0.23 (0.02)	0.64 (0.03)	0.31 (0.01)	0.4 (0.01)	0.20 (0.09)
19–30 y	Males	857	7.1	1.45 (0.17)	0.54 (0.02)	0.48E (0.13)	0.19E (0.04)	0.85 (0.07)	0.31 (0.02)	F	0.05 (0.03)
	Females	992	6.4	1.07 (0.05)	0.57 (0.03)	0.44 (0.04)	0.24 (0.03)	0.58 (0.03)	0.31 (0.02)	F	0.01 (0.01)
31–50 y	Males	2005	15	1.41 (0.06)	0.56 (0.01)	0.53 (0.04)	0.21 (0.02)	0.75 (0.03)	0.30 (0.01)	0.12 (0.01)	0.05 (0.02)
	Females	2384	15.7	1.00 (0.04)	0.54 (0.03)	0.36 (0.02)	0.19 (0.01)	0.55 (0.03)	0.31 (0.01)	0.09 (0.01)	0.05 (0.02)
51–70 y	Males	2151	13.1	1.21 (0.06)	0.52 (0.02)	0.43 (0.04)	0.18 (0.01)	0.71 (0.03)	0.31 (0.01)	0.06E (0.01)	0.02 (0.01)
	Females	2314	13.4	0.99 (0.04)	0.55 (0.03)	0.35 (0.03)	0.20 (0.02)	0.56 (0.02)	0.31 (0.01)	0.04E (0.01)	0.02 (0.01)
≥ 71 y	Males	1180	4.3	1.24 (0.05)	0.60 (0.01)	0.47 (0.03)	0.22 (0.02)	0.73 (0.03)	0.35 (0.01)	F	0.003 (0.001)
	Females	1448	5.4	1.02 (0.05)	0.61 (0.03)	0.37 (0.04)	0.22 (0.02)	0.62 (0.03)	0.38 (0.02)	F	0.004 (0.001)
*Household income*											
< $30,000		3579	15.2	1.13 (0.04)	0.59 (0.02)	0.43 (0.03)	0.22 (0.01)	0.63 (0.02)	0.33 (0.01)	0.04E (0.01)	0.02E (0.004)
$30,000–$49,999		3459	16.4	1.22 (0.04)	0.58 (0.02)	0.46 (0.04)	0.22 (0.02)	0.67 (0.03)	0.32 (0.01)	0.07 (0.01)	0.03 (0.003)
$50,000–$69,999		3005	14.9	1.11 (0.07)	0.54 (0.02)	0.39 (0.04)	0.19 (0.01)	0.64 (0.02)	0.31 (0.01)	F	0.02E (0.01)
$70,000–$99,999		3530	18	1.22 (0.04)	0.57 (0.02)	0.45 (0.03)	0.21 (0.01)	0.69 (0.02)	0.33 (0.01)	0.12 (0.01)	0.06 (0.004)
$100,000–$149,999		3534	20.7	1.25 (0.04)	0.58 (0.02)	0.46 (0.02)	0.22 (0.01)	0.69 (0.03)	0.32 (0.01)	0.1 (0.01)	0.04 (0.003)
> $150,000		2563	14.7	1.21 (0.05)	0.55 (0.02)	0.44 (0.03)	0.20 (0.01)	0.66 (0.02)	0.31 (0.01)	0.5 (0.01)	0.21 (0.004)
*Race*											
Black		394	3.5	0.98 (0.08)	0.49 (0.03)	0.39 (0.05)	0.19 (0.02)	0.47 (0.04)	0.25 (0.02)	F	F
East Asian		759	5.2	0.79 (0.03)	0.44 (0.02)	0.52 (0.05)	0.16 (0.02)	0.45 (0.03)	0.25 (0.01)	F	F
Indigenous (First Nations, Inuk/Inuit, Métis)		906	3.0	1.26 (0.10)	0.61 (0.03)	0.28 (0.02)	0.25 (0.02)	0.65 (0.04)	0.32 (0.02)	F	F
Latin American		203	1.4	1.14 (0.11)	0.57 (0.05)	0.39 (0.06)	0.19 (0.02)	0.68 (0.07)	0.36 (0.05)	F	F
Middle Eastern		316	2.6	1.1 (0.10)	0.51 (0.03)	0.33 (0.04)	0.15 (0.02)	0.70 (0.08)	0.32 (0.02)	F	F
South Asian		677	5.0	0.98 (0.04)	0.5 (0.02)	0.39 (0.03)	0.20 (0.02)	0.50 (0.03)	0.26 (0.01)	F	F
Southeast Asian		496	3.4	0.93 (0.13)	0.46 (0.03)	0.38E (0.08)	0.19 (0.01)	0.49 (0.05)	0.25 (0.01)	F	F
White		15,215	72.1	1.27 (0.02)	0.60 (0.01)	0.46 (0.01)	0.22 (0.01)	0.72 (0.01)	0.34 (0.005)	F	F
Other^a^		704	3.8	1.01 (0.10)	0.56 (0.06)	0.40E (0.09)	0.22E (0.05)	0.49 (0.05)	0.27 (0.02)	F	F

Values followed by an E are flagged as having a large CV (16.6– < 33.3%) and should be interpreted with caution. Values replaced by an F were suppressed as they have a very large CV (> 33.3%) and do not meet Statistics Canada’s quality guidelines

^a^Includes multiple racial identities and not stated

^b^TFA from foods containing a mix of n-TFA and i-TFA (mixed TFA)

**Table 2:** Percentiles of intake of total TFA by people in Canada aged 1 year and older and proportion of the population with intakes below 1% of total daily energy before the prohibition of partially hydrogenated oils

		Distribution of total TFA intakes (% Energy)							% of population with total TFA < 1% of E
		P5	P10	P25	P50	P75	P90	P95	
Total		0.30 (0.01)	0.34 (0.00)	0.43 (0.00)	0.54 (0.00)	0.68 (0.00)	0.83 (0.00)	0.93 (0.01)	96.87
*Age and sex groups*									
1–3 y		0.33 (0.04)	0.39 (0.04)	0.51 (0.04)	0.68 (0.02)	0.87 (0.03)	1.07 (0.06)	1.2 (0.08)	85.85
4–8 y		0.43 (0.05)	0.47 (0.03)	0.54 (0.02)	0.64 (0.01)	0.75 (0.03)	0.87 (0.05)	0.94 (0.07)	97.13
9–13 y	Males	0.49 (0.08)	0.53 (0.07)	0.59 (0.04)	0.67 (0.04)	0.76 (0.07)	0.85 (0.12)	0.91 (0.15)	98.40
	Females	0.41 (0.04)	0.45 (0.03)	0.53 (0.03)	0.63 (0.03)	0.74 (0.05)	0.86 (0.07)	0.94 (0.09)	96.89
14–18 y	Males	0.34 (0.04)	0.38 (0.03)	0.46 (0.02)	0.56 (0.02)	0.69 (0.04)	0.84 (0.07)	0.94 (0.09)	96.86
	Females	0.37 (0.04)	0.41 (0.02)	0.47 (0.02)	0.56 (0.02)	0.66 (0.03)	0.77 (0.04)	0.84 (0.05)	99.05
19–30 y	Males	F	0.30E (0.07)	0.38 (0.06)	0.50 (0.03)	0.65 (0.04)	0.82 (0.13)	0.94E (0.20)	96.52
	Females	0.31E (0.07)	0.35E (0.06)	0.44 (0.05)	0.55 (0.03)	0.68 (0.03)	0.83 (0.05)	0.93 (0.07)	97.20
31–50 y	Males	0.24E (0.04)	0.29 (0.03)	0.38 (0.02)	0.51 (0.01)	0.68 (0.02)	0.87 (0.04)	1.01 (0.06)	94.71
	Females	0.28E (0.07)	0.33 (0.03)	0.41 (0.03)	0.52 (0.03)	0.65 (0.03)	0.80 (0.04)	0.90 (0.05)	97.60
51–70 y	Males	0.26 (0.03)	0.31 (0.03)	0.38 (0.02)	0.49 (0.02)	0.62 (0.03)	0.76 (0.05)	0.86 (0.07)	98.51
	Females	0.31 (0.03)	0.35 (0.03)	0.43 (0.03)	0.53 (0.03)	0.64 (0.04)	0.77 (0.05)	0.85 (0.06)	98.78
≥ 71 y	Males	0.29 (0.04)	0.34 (0.03)	0.43 (0.02)	0.57 (0.01)	0.73 (0.02)	0.9 (0.04)	1.01 (0.06)	94.61
	Females	0.35 (0.05)	0.39 (0.01)	0.48 (0.01)	0.59 (0.03)	0.72 (0.04)	0.86 (0.07)	0.96 (0.08)	96.46
*Household income*									
< $30,000		0.28 (0.03)	0.33 (0.02)	0.42 (0.02)	0.55 (0.02)	0.72 (0.02)	0.91 (0.04)	1.03 (0.06)	94.02
$30,000–$49,999		0.34 (0.05)	0.38 (0.05)	0.46 (0.03)	0.56 (0.02)	0.68 (0.03)	0.80 (0.05)	0.87 (0.07)	98.48
$50,000–$69,999		0.26 (0.04)	0.30 (0.04)	0.39 (0.03)	0.51 (0.02)	0.66 (0.03)	0.82 (0.06)	0.92 (0.09)	97.09
$70,000–$99,999		0.28 (0.03)	0.32 (0.02)	0.41 (0.02)	0.54 (0.02)	0.69 (0.03)	0.87 (0.05)	0.99 (0.07)	95.21
$100,000–$149,999		0.34E (0.07)	0.38 (0.06)	0.46 (0.04)	0.56 (0.02)	0.67 (0.03)	0.80 (0.07)	0.88 (0.09)	98.24
> $150,000		0.26 (0.03)	0.30 (0.02)	0.39 (0.02)	0.52 (0.02)	0.67 (0.02)	0.85 (0.04)	0.98 (0.06)	95.51
*Race*									
Black		0.21E (0.04)	0.25 (0.04)	0.33 (0.04)	0.46 (0.03)	0.61 (0.04)	0.78 (0.08)	0.91 (0.11)	97.01
East Asian		0.20E (0.05)	0.23E (0.04)	0.31 (0.03)	0.41 (0.02)	0.54 (0.03)	0.68 (0.07)	0.78 (0.10)	99.09
Indigenous (First Nations, Inuk/Inuit, Métis)		0.33E (0.06)	0.37 (0.05)	0.46 (0.04)	0.58 (0.03)	0.73 (0.03)	0.89 (0.07)	1.00 (0.10)	94.87
Latin American		0.23E (0.08)	0.28E (0.07)	0.38 (0.06)	0.52 (0.05)	0.71 (0.07)	0.93 (0.13)	1.09 (0.17)	92.40
Middle Eastern		0.21E (0.04)	0.25 (0.04)	0.35 (0.03)	0.47 (0.03)	0.63 (0.05)	0.80 (0.09)	0.92 (0.13)	97.06
South Asian		0.33E (0.09)	0.36E (0.08)	0.41 (0.05)	0.49 (0.02)	0.57 (0.04)	0.66 (0.09)	0.72E (0.13)	99.91
Southeast Asian		0.25E (0.07)	0.28E (0.06)	0.35 (0.05)	0.44 (0.03)	0.55 (0.04)	0.66 (0.07)	0.75 (0.09)	99.54
White		0.33 (0.01)	0.37 (0.01)	0.46 (0.01)	0.57 (0.01)	0.71 (0.01)	0.85 (0.02)	0.95 (0.02)	96.48
Other^a^		0.29E (0.08)	0.33E (0.08)	0.41 (0.06)	0.53 (0.06)	0.67 (0.07)	0.81 (0.12)	0.91E (0.16)	97.45

Values followed by an E are flagged as having a large CV (16.6– < 33.3%) and should be interpreted with caution. Values replaced by an F were suppressed as they have a very large CV (> 33.3%) and do not meet Statistics Canada’s quality guidelines

^a^Includes multiple racial identities and not stated

**Fig. 1:**
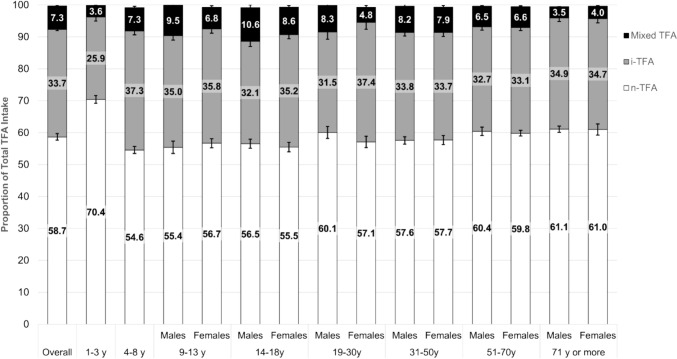
Proportion of total *trans* fat intake coming from foods containing only n-TFA, from foods containing only i-TFA, or from foods containing a mix of n-TFA and i-TFA (mixed TFA) for different age sex-groups in Canada before the prohibition of partially hydrogenated oils. Trans fatty acid (TFA), industrial TFA (i-TFA), natural TFA (n-TFA)

For the overall population, foods containing only n-TFA provided more than half (58.7 [SE 1.1]%) of total TFA intake, while foods containing only i-TFA provided about one third (33.7 [SE 0.4]%) of total TFA (Fig. 1 and Table 1). Compared to other age-sex groups, children aged 1–3 years consumed the highest proportion of total trans fat as n-TFA (70.4 [SE 1.2]%) and the lowest proportion as i-TFA (25.9 [SE 1.3]%).

No meaningful difference in total TFA intake was observed between income groups, with the means varying from 0.54 (SE 0.021) % of energy in the subgroup earning $50,000–$69,999 per year to 0.59 (SE 0.016) % in the subgroup earning less than $30,000 per year (Table 1). The 90th percentile of total TFA intake was below 1% of energy in all income subgroups, and 95th percentile below in all except those earning < $30 000 per year (1.03 [SE 0.06] %En) (Table 2). In all income groups, ≥ 57.7 (SE 1.0)% of total TFA came from foods containing only n-TFA, and about one third of total TFA intake was provided by i-TFA, the latter ranging from 32.4 (SE 1.0)% in the $50,000–$69,999 group to 34.7 (SE 1.1)% in the $30,000–$49,999 group (Supplemental Fig. 2A).

**Fig. 2:**
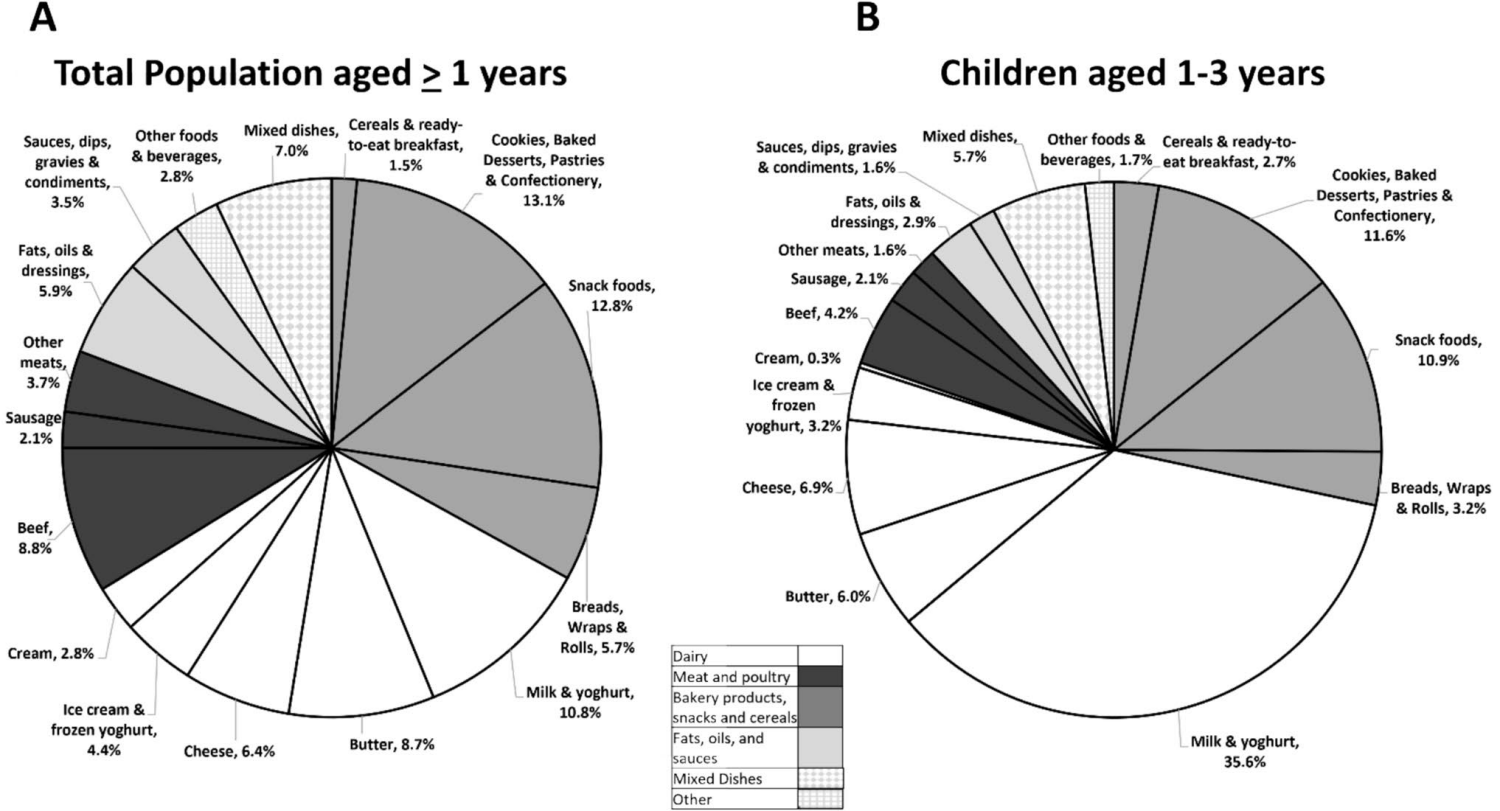
Contribution (% total TFA intake) of Food Categories to Total TFA Intakes in CCHS 2015 Nutrition in all age-sex groups (**A**) and children aged 1–3 (**B**). Taken separately, each of the food categories comprised in ‘Other foods & beverages’ (soups, eggs, nuts & seeds, coffee whiteners, hot chocolate without milk, egg noodles, fast food hashed brown potatoes) contributed to < 1% of total TFA intake. The contribution of the food-subcategories included in the main categories depicted here is detailed in Supplemental Table 1

With respect to race, all subgroups had mean and 90th percentile intakes ≤ 1%En, with those who self-identified as East or Southeast Asian reporting the lowest mean intake (0.44 and 0.46%En, respectively), and those identifying as Indigenous or White reporting the highest mean intakes (0.61 and 0.60%En, respectively) (Tables 1, 2). Proportions of TFA provided from natural and industrial sources were similar to the total population in all race subgroups. Most TFA were provided by foods containing only n-TFA, ranging from 53.7 (SE 2.4)% of total TFA in the South Asian subgroup, to 62.3 (SE 3.4)% of total TFA intake in the Latin American subgroup (Supplemental Fig. 2B). In all race groups, about one third of total TFA intake was provided by i-TFA, ranging from 30.9 (SE 2.6)% in the Middle Eastern subgroup to 37.0 (SE 2.9)% in the Southeast Asian subgroup (Supplemental. Fig. 2B).

### Main contributors to total TFA intake and to intake of TFA from different sources

For the general population ≥ 1 year, n-TFA from dairy foods accounted for one third of total TFA intake, with milk and yoghurt providing 10.8% of total TFA, followed by butter at 8.7% and cheese with 6.4% of total TFA (Fig. 2A). Meats contributed to 14.6% of total TFA intake, over half of which (8.8%) came from beef. About one third (31.6%) of total TFA intake in the overall population came from foods traditionally known to be sources of i-TFA, such as cookies, baked desserts, pastries, confectionery, snack foods, breads, wraps, and rolls (Fig. 2A). Altogether, cookies, baked desserts, pastries and confectionery provided 13.1% of total TFA intake, snack foods contributed to another 12.8% and breads, wraps and rolls contributed 5.7%. In children aged 1–3 years, the only age-sex group who did not meet Health Canada’s target of ≥ 90% of the population consuming less than 1%En as total TFA, milk and yogurt accounted for 35.6% of total TFA, and other dairy products to a further 16.4% (Fig. 2B).

**Fig. 3:**
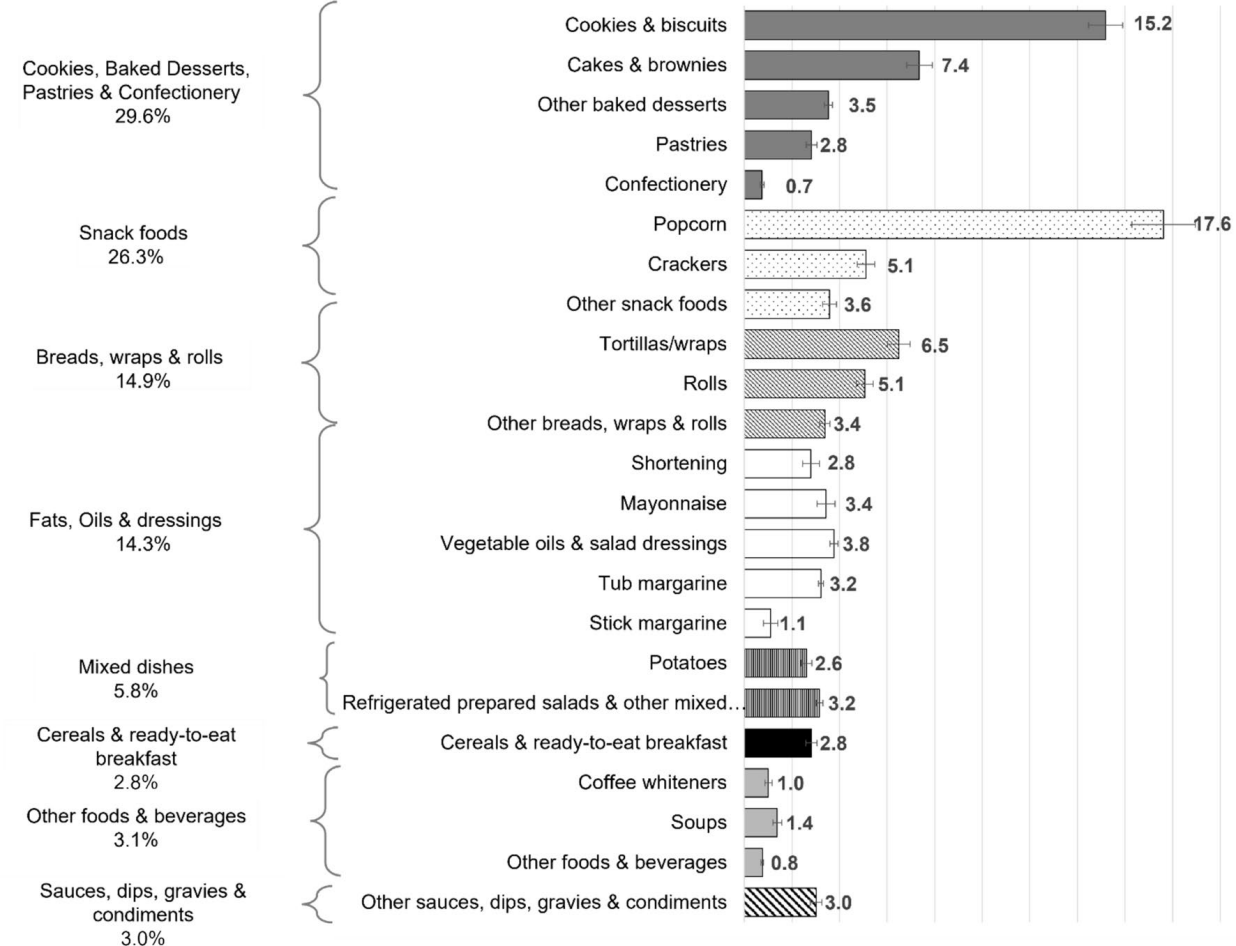
Contribution of foods containing only i-TFA to total i-TFA intake by people in Canada aged 1y and older before the prohibition of partially hydrogenated oils

Because the prohibition on PHO targets i-TFA, there was an interest in identifying the relative contributions of food categories to total i-TFA intakes. Baked goods were the largest contributors to total i-TFA intakes (29.6 [SE 0.9]%), followed by snack foods like popcorn and crackers (26.3 [SE 1.3]%), breads, wraps and rolls (14.9 [SE 0.6]%), fats, oils and dressings (14.3 [SE 0.6]%), mixed dishes (5.8 [SE 0.3]%), cereals and ready-to-eat breakfasts (2.8 [SE 0.2]%), other foods and beverages like soups and coffee whiteners (3.1 [SE 0.2]%), and sauces, dips, gravies and condiments (3.0 [SE 0.2]%) (Fig. 3).

## Discussion

The data used for this analysis was collected after Canada had already taken several measures to decrease intakes of total TFA, but had not yet implemented a ban on PHO [22–24]. In the 2000s, Canada became the first country to implement mandatory labelling of *trans* fats on prepackaged food, established voluntary targets for TFA content in foods supported by a monitoring and reporting program, and initiated various consumer awareness and education initiatives [25, 26]. In this analysis of 2015 dietary intake data, we show that average intakes of total TFA were below the 1%En threshold recommended by the WHO prior to the addition of PHO to Canada’s List of Contaminants and Other Adulterating Substances in Foods, which banned this source of TFA from the food supply [27]. These results are consistent with Canadian national biomonitoring data on red blood cell fatty acids from a similar time period, which also reported levels of TFA below a proposed risk threshold of 1% of fatty acids [28].

The average intakes of total TFA calculated from the 2015 dietary intake data and updated food composition data (0.57%En) are lower than reported previously for Canada from 1995 (3.7%En), 2004 (2.0%En), and 2008 (1.4%En) [5, 29]. The 2015 CCHS Nutrition survey had some key differences from previous surveys, including incorporation of a second 24-h recall, lower sample size and response rate, and use of an updated food and nutrient database. Total fat accounted for a similar %En in CCHS 2004 and 2015 (2015—Males 19 + : 32.3 [SE 0.2], females 19 + : 32.2 [SE 0.2]; 2004—Males 19 + : 31.5 [SE 0.2], Females 19 + : 31.2 [SE 0.2]) [30]. While there are methodological differences between these surveys, this suggests that the reduction in intakes of total TFA over time is not explained by a lower estimated intake of total fat. Two systematic reviews of national consumption data globally have reported either stable or decreased intakes of total TFA over time, with decreases most prominent in countries that implemented voluntary or mandatory measures to reduce the TFA content of foods, which is in line with our results [31, 32]. Additionally, a systematic review examining the global impact of TFA reduction policies found that voluntary TFA limits were associated with reductions in intake ranging from 20–38%, while mandatory labelling led to reductions of 30–74% [33]. Together, these findings suggest that the observed reductions in TFA intakes in Canada are consistent with the range of outcomes reported in other countries with similar policies. Our data suggest that the multi-faceted approach implemented by Health Canada, along with changes in the global food landscape that may have occurred during this period, were effective at reducing total TFA intakes below the WHO recommended level prior to the prohibition of PHO.

Children aged 1–3 years were the only subgroup examined (including groups by DRI age-sex categories, income, and race) that did not have usual total TFA intakes meeting Health Canada’s target (below 1%En in ≥ 90% of the population) [9]. Previous analyses in 2004 and 2008 found that total TFA intakes as a percent of energy were similar or slightly lower in children aged 1–3 years compared to older children, but were higher than those of adults [29, 34]. The larger relative decreases in intake among other age-sex groups may be explained by a reduction in total TFA intakes over this period driven by product reformulations to reduce i-TFA content following Canada’s multi-faceted approach [7]. As intakes of total TFA drop, n-TFA from ruminant sources can become the main contributor to intakes, and young children in Canada have the highest rate of milk consumption of all age groups [3, 35]. The 90th percentile of total TFA intake in this group (1.07 [SE 0.061] %En) was not substantially higher than the WHO threshold, so it is not clear whether this value should be of public health concern. The WHO threshold of 1%En from total TFA is intended to apply to those ≥ 2 years of age, however the systematic review of RCTs and prospective cohort studies conducted to update its guidance in 2023 identified no studies meeting the inclusion criteria in children, so results were extrapolated from adults [3]. More research is needed to determine whether TFA intakes ≥ 1%En are associated with negative health outcomes in very young children, particularly as over 70% of the total TFA consumed in this population came from natural sources, primarily dairy products.

Although the WHO recommendations apply to all sources of TFA, many public health measures, including Canada’s prohibition of PHO and the WHO’s REPLACE action package, focus on i-TFA [3, 4, 8]. The WHO estimates that n-TFA intakes from ruminant sources are generally low, but that their relative contributions to total intakes may increase as sources of i-TFA are phased out of the food supply [3]. This appears to be the case in Canada, where n-TFA accounted for over half of total TFA intakes. Previous systematic reviews and meta-analyses that have examined the associations between n- and i-TFA and non-communicable diseases have reported a positive association of CHD risk and mortality with intakes of i-TFA, but not n-TFA, however some studies suggest that both sources may have similar effects on blood cholesterol profiles [36–39]. In its systematic reviews, the WHO noted that most studies identified reported results for total TFA intake, and concluded that there was insufficient evidence to support different recommendations for i- and n-TFA sources [3]. Further research is needed to determine whether n- and i- TFA have differing effects on disease risk, particularly in the context of low total TFA intakes.

Strengths of this study include that data was collected from a large, nationally representative survey, and that a subset of dietary intakes were assessed using two 24-h recalls, which enabled estimation of usual intake. This study also has several limitations. People living in remote areas of Canada’s north were identified in the 2012 risk assessment as particularly vulnerable to higher TFA intakes, but the CCHS 2015 did not collect data from people living in the territories. Other studies in these populations, such as the Nunavik Inuit Health Survey 2017 (Qanuilirpitaa?) are important for filling these gaps [40]. Our analysis also provided national, as opposed to provincial or regional estimates of intake, so it is possible that there may be specific locations or communities in Canada that still have elevated intakes that were not identified with our study. In addition, although measures were taken to minimize and correct for misreporting, intake data was self-reported, which could contribute to error in estimates. Further, while efforts were made to develop a comprehensive database of the *trans* fat content of foods, label data had to be used for certain products. Canadian regulations require *trans* fats to appear on food labels, but these values may be rounded to 0 or to the nearest 0.1, 0.5 g or 1 g depending on the TFA and saturated fat content of the product [41]. While comparisons between analytical and labelled TFA contents suggest that differences are negligible, this rounding could represent a source of error in our analyses [42]. Finally, quantitative data on human milk intake was unavailable in the 2015 CCHS Nutrition, so intakes of TFA or energy from human milk were excluded from the calculations of usual intake. Mature human milk collected in Canada in 2011 contained 1.9 (SD 0.5) % of total fat as TFAs (mature human milk is estimated to contain 3.4 [2 SD 1.6–5.2] g total fat / 100 mL) [43, 44]. This could lead to underestimation of TFA intakes particularly within the 1–3 age group, as Canada’s public health guidance recommends breastfeeding to age 2 and beyond [45].

## Conclusion

The results of this study indicate that Canada had already met its target of having total TFA intakes below 1%En in ≥ 90% of the population in 2015, prior to the introduction of the prohibition on PHO. This was true in all DRI age-sex groups except children aged 1–3 years, and in all subgroups of income and race examined. In the Canadian context where diets contained < 1%En as TFA, natural sources of *trans* fats accounted for the majority of total TFA intake. While the prohibition of PHOs has anchored the benefits achieved through earlier policy interventions, future research is needed to determine whether it will lead to further reductions in total TFA intake.

## Supplementary Information

Below is the link to the electronic supplementary material.

Supplementary Material 1.

## Data Availability

Data described in the manuscript, code book, and analytic code will be made available upon request pending application and approval by Statistics Canada and Health Canada.

## Abbreviations

%En: % Of total daily energy
CCHS: Canadian Community Health Survey
i-TFA: *Trans* fatty acids of industrial origin
n-TFA: Naturally occurring *trans* fatty acids
PHO: Partially hydrogenated oils
TFA: *Trans* fatty acids
