# Supplementary material for: Dietary intakes of trans fatty acids before the prohibition of partially hydrogenated oils in Canada
Source: Eur J Nutr. 2024 Dec 30;64(1):59. doi: 10.1007/s00394-024-03569-7 (PMC11685241; doi:10.1007/s00394-024-03569-7)

Supplemental Table 1: Food Categories and Subgroups used for analysis, and proportion of total TFA intake by category subgroups in the total population aged ≥1 year.

Main Food Categories	Category Subgroups	Proportion (SE) of Total TFA Intake
	Beef, not ground	2.5 (0.2)
**Beef**	Ground beef	6.3 (0.3)
	Total Beef	**8.8 (0.3)**
	Rolls	1.9 (0.1)
**Breads, Wraps & Rolls**	Tortillas/wraps	2.3 (0.2)
	Other breads, wraps & rolls	1.4 (0.1)
	Total Breads, wraps and rolls	**5.7 (0.2)**
**Butter**	***Butter***	**8.7 (0.4)**
**Cereals & ready-to-eat breakfast**	***Cereals & ready-to-eat breakfast***	**1.5 (0.1)**
	Cheddar cheese	4.2 (0.1)
**Cheese**	Other cheese	2.2 (0.1)
	Total Cheese	**6.4 (0.2)**
	Cakes & brownies	3.8 (0.2)
	Confectionery	1.4 (0.2)
	Cookies & biscuits	5.5 (0.3)
**Cookies, Baked Desserts, Pastries & Confectionery**		
	Pastries	1.1 (0.1)
	Other baked desserts	1.3 (0.1)
	Total Cookies, Baked Desserts, Pastries & Confectionery	**13.1 (0.4)**
**Cream**	***Cream***	**2.8 (0.2)**
	Mayonnaise	1.4 (0.1)
**Fats, oils & dressings**		
	Shortening	1.4 (0.1)

Main Food Categories	Category Subgroups	Proportion (SE) of Total TFA Intake
	Stick margarine	0.5 (0.1)
	Tub margarine	1.2 (0)
	Vegetable oils & salad dressings	1.2 (0.1)
	Other fats, oils & dressings	0.2 (0)
	Total fats, oils and dressings	**5.9 (0.2)**
	Frozen yoghurt	0.3 (0)
**Ice cream & frozen yoghurt**	Ice cream	4.2 (0.2)
	Total ice cream & frozen yoghurt	**4.4 (0.2)**
	1% M.F. milk	1.4 (0)
	2% M.F. milk	5.1 (0.1)
	3.25% M.F. milk	3.2 (0.2)
**Milk & yoghurt**		
	Yoghurt	0.9 (0)
	Other milks	0.2 (0)
	Total milk & yoghurt	**10.8 (0.2)**
	Mixed dishes with cheese or beef	1.1 (0.1)
	Pizza & sandwiches	3 (0.2)
**Mixed dishes**	Potatoes	1.5 (0.1)
	Refrigerated prepared salads & other mixed dishes	1.5 (0.1)
	Total Mixed Dishes	**7 (0.2)**
	Coffee whiteners	0.4 (0.1)
	Eggs	0.7 (0.1)
**Other foods & beverages**		
	Soups	0.8 (0.1)
	Other foods & beverages	0.8 (0)
	Total other foods & beverages	**2.8 (0.1)**
**Other meats**	Chicken	1.7 (0.1)

Main Food Categories	Category Subgroups	Proportion (SE) of Total TFA Intake
	Pork	1 (0.1)
	Chicken	0 (0)
	Other meats, fish & shellfish	1 (0.1)
	Total other meats	**3.7 (0.2)**
**Sauces, dips, gravies & condiments**	Cream Sauce	2.2 (0.2)
	Other sauces, dips, gravies & condiments	1.3 (0.1)
	Total sauces, dips, gravies & condiments	**3.5 (0.2)**
**Sausage**	Beef sausage	1 (0.1)
	Sausages made of other meats & mixed meat sausages	1.1 (0.1)
	Total sausages	**2.1 (0.1)**
**Snack foods**	Crackers	1.9 (0.1)
	Popcorn	7.4 (0.6)
	Snack puddings	1.8 (0.2)
	Other snack foods	1.6 (0.1)
	Total snack foods	**12.8 (0.6)**

Supplemental Figure 1: Participant Flow Diagram

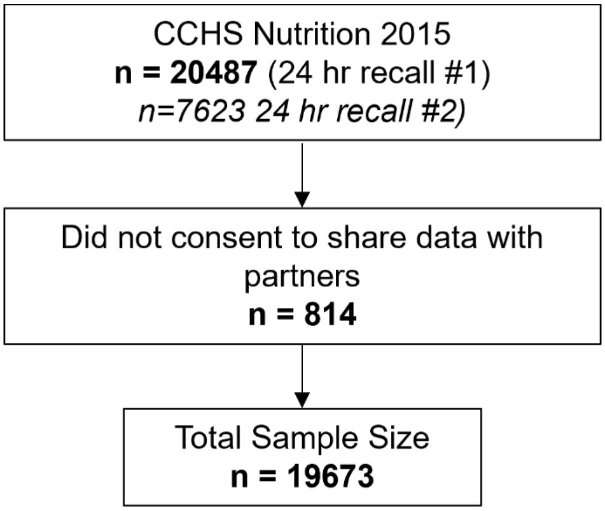

Supplemental Figure 2. Proportion of total *trans* fat intake coming from foods containing only n-TFA, from foods containing only i-TFA, or from foods containing a mix of n-TFA and i-TFA (mixed TFA) by income (A) and race (B) in Canada before the prohibition of partially hydrogenated oils. Trans fatty acid (TFA), industrial TFA (i-TFA), natural TFA (n-TFA).

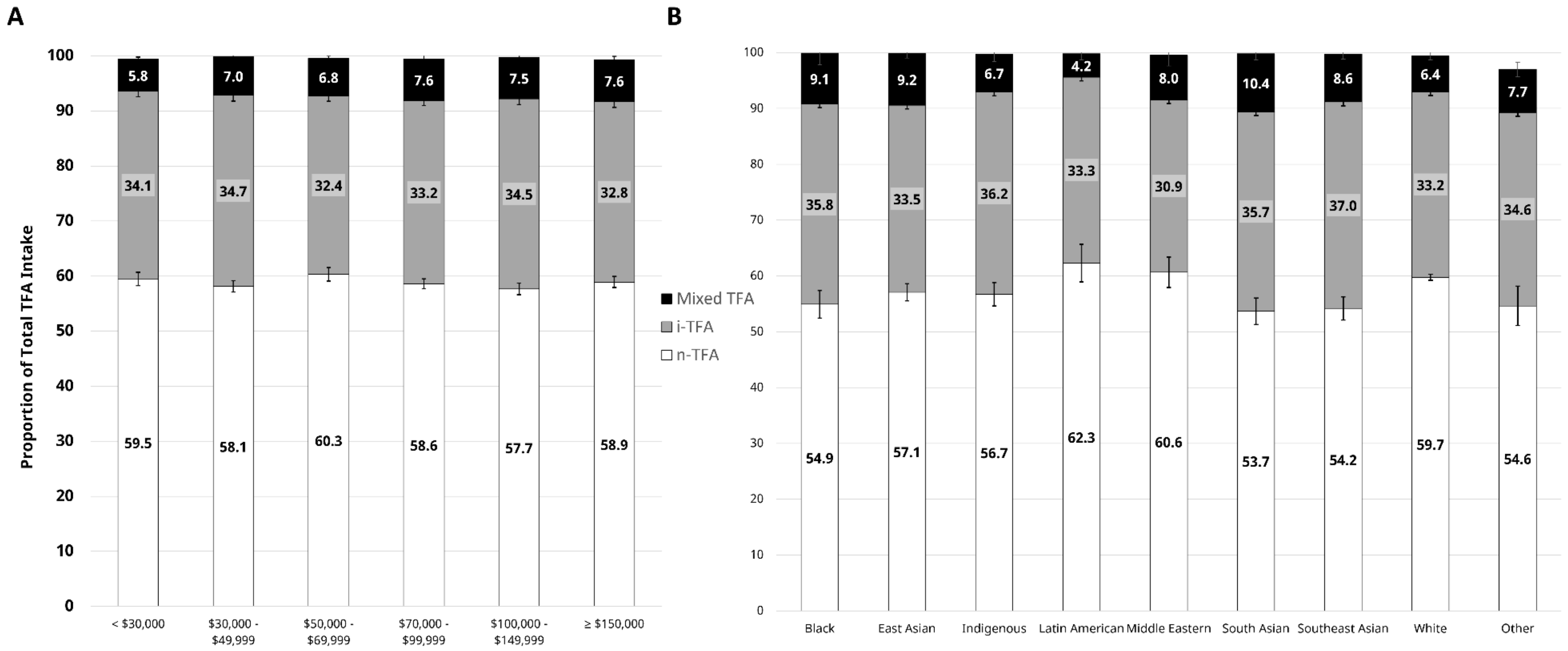

Supplement: Supplementary file 1 — Supplementary Material 1. [file 394_2024_3569_MOESM1_ESM.pdf]
